# Screening variability and change of soil moisture under wide-ranging climate conditions: Snow dynamics effects

**DOI:** 10.1007/s13280-014-0583-y

**Published:** 2015-01-09

**Authors:** Lucile Verrot, Georgia Destouni

**Affiliations:** Department of Physical Geography and Quaternary Geology, Stockholm University, 106 91 Stockholm, Sweden

**Keywords:** Soil moisture, Groundwater, Snow dynamics, Climate change, Landscape scale, Hydrological basin

## Abstract

Soil moisture influences and is influenced by water, climate, and ecosystem conditions, affecting associated ecosystem services in the landscape. This paper couples snow storage-melting dynamics with an analytical modeling approach to screening basin-scale, long-term soil moisture variability and change in a changing climate. This coupling enables assessment of both spatial differences and temporal changes across a wide range of hydro-climatic conditions. Model application is exemplified for two major Swedish hydrological basins, Norrström and Piteälven. These are located along a steep temperature gradient and have experienced different hydro-climatic changes over the time period of study, 1950–2009. Spatially, average intra-annual variability of soil moisture differs considerably between the basins due to their temperature-related differences in snow dynamics. With regard to temporal change, the long-term average state and intra-annual variability of soil moisture have not changed much, while inter-annual variability has changed considerably in response to hydro-climatic changes experienced so far in each basin.

**Electronic supplementary material:**

The online version of this article (doi:10.1007/s13280-014-0583-y) contains supplementary material, which is available to authorized users.

## Introduction

Soil moisture is a dynamic variable of major importance in the hydrologic cycle (Corradini 2014) and for a range of different climate, environmental, and societal conditions (Seneviratne et al. 2010). It also affects ecosystem services and water connectivity in the landscape including, for example, the occurrence and the connectivity of wetlands and their ecosystem services (Kininmonth et al. 2015; Moor et al. 2015; Quin et al. 2015).

Soil moisture (referring to the amount of water stored) over some given soil depth varies temporally depending on the seasonality and fluctuations of hydro-climate at the surface (Rodriguez-Iturbe et al. 1991) as well as of the groundwater table position in the subsurface (Destouni and Verrot 2014). It varies spatially depending on several factors. These include local hydro-climatic conditions, topography and vegetation at the surface, and soil type. The spatial variation of soil moisture due to soil type is a result of both characteristic soil hydraulic property averages and variability in these properties in the subsurface (Destouni 1993; Russo 1998).

Models of soil moisture have focused on different aspects of its full complexity depending on study question and application. The near-surface temporal variability of soil moisture has been in focus primarily in energy balance and climate-related studies, whereas water resource and quality studies have considered greater soil depths including groundwater conditions, which has required stronger focus on the whole vadose zone and its coupling with groundwater. In a recent development, these unsaturated zone and saturated zone aspects of soil moisture have been coupled in an analytical modeling framework, which should be useful for at least first-order quantification of long-term and large-scale variability and change of average soil water content in a changing climate, considering also the associated variability and change of groundwater table conditions (Destouni and Verrot 2014).

In various numerical climate and watershed models, snow storage and melting processes, and their link to climatic variables—typically temperature and precipitation—and consequences for hydrological conditions, like runoff, are widely addressed, either with simple models (Molini et al. 2011) or analytically (Schaefli et al. 2013). As other hydrological variables, soil moisture is also related to snow storage and melting conditions (Bosson. et al. 2012), but this is more rarely taken into account in relatively simple analytical approaches to soil moisture in a changing climate.

**Fig. 1:**
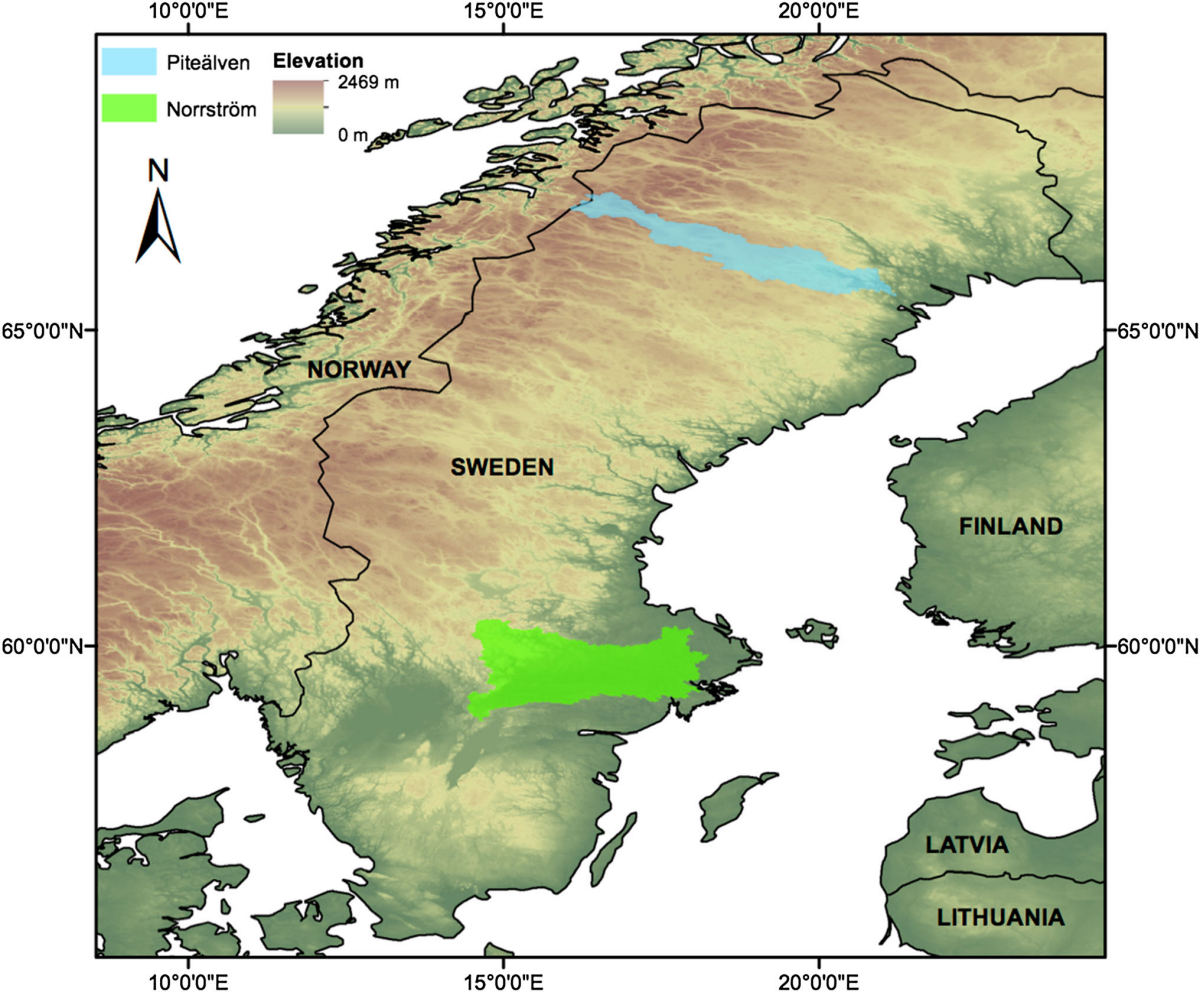
Location of the two study basins: Norrström in *green* and Piteälven in *blue*. The base layer is a Digital Elevation Model provided by the European Environment Agency (EEA 2012)

The present study addresses and aims at bridging this gap in analytical soil moisture modeling by extending the framework developed by Destouni and Verrot (2014) to introduce a model which takes into account more widely different hydro-climatic conditions, including such where snow storage and melting effects are important. This extension is needed to enable assessment of differences between hydrological basins at climatically different locations, as well as temporal changes in soil moisture across a wider range of different hydro-climatic conditions. The extended snow-accounting modeling framework is here further applied to region-specific quantification and spatial comparison of soil moisture development under observed historic-to-present hydro-climatic conditions during the twentieth and early twenty-first century in two climatically different Swedish hydrological basins: the Norrström drainage basin, located in the central-southeastern part of Sweden, and the Piteälven basin, located in the northern part of the country (Fig. 1). The main question addressed by each regional assessment and the spatial comparison is how soil moisture is affected by temporal change and spatial differences in hydro-climatic conditions, considering in particular the different snow storage and melting conditions of the two investigated basins. In this context, soil moisture is quantified in terms of the average state of volumetric soil water content at basin-scale and over some given depth of interest.

## Materials and methods

### Site characteristics

This study concretizes, exemplifies, and compares results for soil moisture in the two climatically different basins of Norrström and Piteälven (Fig. 1). In Norrström (22 650 km^2^), which has been hydrologically well investigated and described in more detail in previous studies (Destouni et al. 2013; Jaramillo et al. 2013; Destouni and Verrot 2014), the long-term average annual temperature is 5.8 °C, the average annual precipitation is 600 mm, and the average annual runoff is 225 mm over the entire present study period of 1950–2009.

The Piteälven basin (10 817 km^2^) has also been investigated and described in previous studies (Humborg et al. 2004; Aldahan et al. 2006), including comparisons with Norrström with regard to their hydro-climatic conditions and changes (Destouni et al. 2013), however, not before with specific regard to soil moisture. The long-term average annual temperature is here −0.8 °C, the average annual precipitation is 584 mm, and the average runoff is 468 mm for the whole period 1950–2009.

The Piteälven basin is thus subject to considerably colder conditions than Norrström. This difference may play an important role in winter, when the precipitation falls mainly as snow in Piteälven, whereas in Norrström, it may still largely fall as rain. In Piteälven, the winter precipitation is then to a larger degree than in Norrström stored as snow at the surface and does not contribute to soil moisture before it melts when the weather gets warmer in spring. The present extension of analytical soil moisture modeling to also include snow storage and melting dynamics, as described in the following section, may thus be necessary for direct comparison of soil moisture variability and change in such different climatic conditions as in these two basin examples.

### Modeling approach

We follow the previously developed analytical modeling framework by Destouni and Verrot (2014). For calculation of water content *θ*_*uz*_ [–] in the unsaturated zone, average water content *θ*_*z*_ [–] over a fixed soil depth *z* [L] from the surface, and groundwater level *z*_gw_ [L] within *z*, novel extensions are made here from the basic framework of Destouni and Verrot (2014) in order to account for a wider range of hydro-climatic conditions, including snow dynamics.

One main model extension made for the calculation of *θ*_*uz*_ [Electronic Supplementary Material (ESM) section Methods] is in order to account for the fact that not the whole observed runoff *R* [LT^−1^], but only some fraction of it (denoted *γ* [–]), actually flows through the soil-groundwater system where it can contribute to soil moisture. An effective runoff measure *R*_eff_ [LT^−1^] is here used to approximate average vertical soil water flux through the unsaturated zone (*q* [LT^−1^]) in equation S2 of ESM—Methods, and the fraction *γ* relates effective runoff *R*_eff_ [LT^−1^] to measured runoff *R* as *R*_eff_ = *γR* with 0 ≤ *γ*≤1.

Previous studies have shown that the soil water flux *q* and its temporal variability can successfully be estimated for such *θ*_*uz*_ estimation from available time series of the contribution of water flow through the soil to runoff *R* (Destouni 1991, 1993). Use of *R*_eff_ in this estimation implies averaging over the (basin, watershed, catchment, field) area that is integrated by the flow that feeds into *R* through the soil-groundwater system. Such simplified area-depth-averaged expression of soil water content *θ*_*uz*_ in the unsaturated zone has been tested and found practically useful by both numerical experimentation (Destouni 1991) and field experimentation (Graham et al. 1998) over different soil depths and different time scales of averaging *q* ≈ *R*_eff_.

On annual average basis, *γ* is typically above 0.5 and in many cases close to 1 for a wide range of investigated temperate, through cold, to permafrost region conditions (Bosson. et al. 2012). However, differences in relevant *γ* values between basins may still be important and can then readily be accounted for when comparing different hydro-climatic conditions, as in the present study.

The approach to estimating unsaturated water content *θ*_*uz*_ by use of *q* ≈ *R*_eff_ also implicitly (through actual *R* observation data) and explicitly (through *γ* dependence on temperature) accounts for snow storage-melting dynamics effects. Specifically, for a given month in a cold period, the precipitation that falls and is stored as snow does not contribute to the observed *R*, whereas the water added to the soil by snow melting during warmer months does contribute to the observed *R*, in addition to the water amount that comes directly from liquid precipitation minus evapotranspiration. By expressing the unsaturated water content *θ*_*uz*_ as a function of *q* ≈ *R*_eff_ and relevant depth-averaged soil parameters (equation S2 in ESM—Methods; see also ESM section Data regarding soil data used to evaluate these parameters), the need for model extension in order to account for snow-ice dynamics effects is limited to modeling of the fraction *γ* dynamics, since the measured *R* dynamics already reflect such effects.

A second main extension made here to the model of Destouni and Verrot (2014) is to explicitly consider effects of snow storage-melting dynamics on the change in soil water storage (Δ*S* [LT^−1^] expressed as volume of water per unit area and unit time). This extension introduces an effective precipitation of liquid water *P*_eff_ [LT^−1^], which relates to measured precipitation *P* [LT^−1^] as described further below. The storage change is then at any point in time given by the water balance expression Δ*S* = *P*_eff_ – *ET* − *R*_eff_, with *ET* [LT^−1^] being evapotranspiration, with resulting net cumulative change in water storage *S*(*t*;*t*_0_) from some initial time *t*_0_ to time *t* becoming

1 $$ S(t;t_{0} ) = \int\limits_{{t_{0} }}^{t} {\{ \gamma [P_{\text{eff}} (\tau ) - ET(\tau )] - R_{\text{eff}} (\tau )\} } \,{\text{d}}\tau, $$

where *τ* is a dummy integration variable, and the factor *γ* comes in also here to distribute to the soil a proportional fraction of water from total *P*_eff_ − *ET* as the fraction *γ* of total *R* flowing through the soil-groundwater system. The associated change in the depth of the groundwater table can further be estimated by distributing the storage change Δ*S* at each time point over the available unsaturated pore space per unit area (*θ*_s_ − *θ*_*uz*_) and integrating the result from initial time *t*_0_ to time *t* as

2 $$ z_{\text{gw}} (t;t_{0} ) = z_{{{\text{gw}} - 0}} (t_{0} ) + \int\limits_{{t_{0} }}^{t} {\frac{{\gamma [P_{\text{eff}} (\tau ) - ET(\tau )] - R_{\text{eff}} (\tau )}}{{\theta_{\text{s}} - \theta_{uz} (\tau )}}} \,{\text{d}}\tau, $$

where *z*_gw−0_ is the initial groundwater level position at time *t*_0_. The average water content *θ*_*z*_ over the whole considered soil depth *z* can thus finally be obtained as

3 $$ \theta_{z} (t) = \frac{{z_{\text{gw}} (t)\theta_{uz} (\tau ) + \left( {z - z_{\text{gw}} (t)} \right)\theta_{\text{s}} }}{z} $$

The effective precipitation *P*_eff_ is used in Eqs. 1 and 2 because only the liquid water part of *P*, *P*_water_ [LT^−1^], in addition to a snow-melt contribution, *S*_M_ [LT^−1^], can effectively contribute to changes in water storage and in the depth of the groundwater table. The *P*_eff_ value is then obtained from a simple snowpack model proposed by Rankinen et al. (2004a), based on a degree-day conceptualization. Such models have been developed (Vehviläinen 1992; Tobin et al. 2013) and widely used for different regional conditions (Braithwaite and Zhang 2000; Tobin et al. 2011), including for Scandinavia (Mörth et al. 2007; Juston et al. 2009).

The modeling approach of Rankinen et al. (2004a) was developed within the frame of the Integrated Nitrogen Model for Catchments (INCA) model and tested for conditions in Finland (Limbrick et al. 2000; Granlund et al. 2004; Rankinen et al. 2004b). Following this approach, the manifestation of measured precipitation *P* as snow (*P*_snow_) or liquid water rainfall (*P*_water_ = *P* − *P*_snow_) is at any point in time first determined on the basis of mean air temperature *T*_A_ [Θ] as

4a $$ P_{\text{snow}} = 0;\quad {\text{for}}\;T_{\text{A}} \ge T_{\text{U}} $$

4b $$ P_{\text{snow}} = \frac{{P(T_{\text{U}} - T_{\text{A}} )}}{{T_{\text{U}} - T_{\text{L}} }};\quad {\text{for}}\;T_{\text{L}} \le T_{\text{A}} \le T_{\text{U}} $$

4c $$ P_{\text{snow}} = P;\quad {\text{for}}\;T_{\text{A}} \le T_{\text{L}} $$

with *T*_U_ [Θ] and *T*_L_ [Θ] being temperature thresholds, above and below which precipitation is considered to fall entirely as water (Eq. 4a) or as snow (Eq. 4c), respectively. Furthermore, Eq. 4b states that when the air temperature is between *T*_L_ and *T*_U_, the precipitation falls partly as water and partly as snow. If *T*_A_ is greater than *T*_M_ [Θ], with the latter being the temperature at which the snow starts to melt, the associated flux of meltwater *S*_M_ is determined as

5 $$ S_{\text{M}} = (T_{\text{A}} - T_{\text{M}} )F_{\text{M}},$$

where *F*_M_ [LT^−1^ Θ^−1^] is a degree-day factor for snow melt.

From the above temperature conditions, *P*_eff_ in Eqs. 1 and 2 can be calculated as

6 $$ P_{\text{eff}} = P_{\text{water}} + S_{\text{M}} $$

The original model presented by Rankinen et al. (2004a) accounts also for evaporation from the snow. Here, however, the effects of basin-scale evapotranspiration, which includes evaporation in addition to transpiration, are explicitly accounted for in Eqs. 1 and 2 based on actual observed hydro-climatic data along with basin-scale water balance constraints, as described further in the next section.

### Data

To obtain concrete regional evaluation results from the above quantification framework, daily values of *R* from 1901 until 2010 were used, as downloaded from the Swedish Meteorological and Hydrological Institute (SMHI) website (SMHI 2010), for the Övre station in the Norrström basin. For the Piteälven basin, only monthly *R* values were available from 1928 until 2013 and these were similarly used and downloaded for the Sikfors station. We further used as model inputs the time series of observed daily *P* and *T* from the E-OBS dataset of the EU-FP6 project ENSEMBLES (Haylock et al. 2008) with a 0.25° × 0.25° resolution from 1950 until 2013.

Effective runoff *R*_eff_ was further calculated by use of reported simulated *γ* factors for different hydro-climatic and landscape conditions in typical Swedish soils (Bosson et al. 2012). Specifically, *γ* was here calculated based on the Bosson et al. simulation results for the ratio of groundwater recharge (*R*_gw_ in Bosson et al. 2012) to measured total runoff *R*. On average, this ratio was found to be 0.73 for temperate and 0.53 for cold (but without permafrost) conditions. We used then here, for exemplification of *γ* dynamics effects, a *γ* value of 0.53 for months with negative average temperature (when both *R* and *γ* are relatively small due to snow storage and frozen ground conditions) and a *γ* value of 0.73 for months with positive average temperature (when both *R* and *γ* are relatively large due to snow melt and unfrozen ground conditions).

The parameter values of *T*_U_, *T*_L_, *T*_M_, and *F*_M_, required for calculation of *P*_eff_, were taken from Rankinen et al. (2004b). Based on comparable climate characteristics, the parameters for the Norrström basin and the Piteälven basin were assumed to be similar to those used in Rankinen et al. (2004b) for the observation station 1201 and the observation station 7501, respectively. Both stations are located in Finland, one in the southern and one in the northern part. The coordinates of the station 1201 are (60°49, 23°30). The mean annual precipitation is 607 mm per year, and the mean annual temperature is 4.3 °C. For the station 7501, the coordinates are (67°22, 26°37), the precipitation is 507 mm per year on average, and the mean annual temperature is −0.8 °C. The values for *T*_L_ and *T*_U_ were chosen as the mean values of the ranges presented by Rankinen et al. (2004b). The used values for the present two study basins are listed in Table 1.

**Table 1:** Values of the parameters *T*_M_ (temperature threshold for snow melt), *T*_L_ (temperature threshold, below which the precipitation is considered to fall entirely as snow), *T*_U_ (temperature threshold, above which the precipitation is considered to fall entirely as water), and *F*_M_ (degree-day factor for snow melt) for Norrström and for Piteälven (selected values from Rankinen et al. 2004b)

Parameter	Norrström	Piteälven
*T*_M_ (°C)	0.30	0.15
*T*_L_ (°C)	−2.02	−4.00
*T*_U_ (°C)	3.70	0.00
*F*_M_ (mm day^−1^ °C^−1^)	3.34	2.85

Calculated daily values of *P*_eff_ were further aggregated on a monthly basis, in order to be comparable and used together with the available monthly values of *R*_eff_ in the calculations of water contents *θ*_*uz*_ and *θ*_*z*_, according to equations S2 in ESM—Methods and Eq. 3, respectively. With regard to time scales, *P* and *P*_eff_ differ at both daily and monthly resolutions. Annually aggregated values of *P* and *P*_eff_, however, are essentially the same over a hydrological year, i.e., from September to August, as the snow during 1 year commonly also melts during the same year.

Estimation of monthly *ET* values, corresponding to the monthly *P*_eff_ and *R*_eff_ values, is further required in Eq. 2. These monthly *ET* values were estimated for the whole investigation period 1950–2009 based on annual values of directly observed *ET* over the time period 2000–2010 (ORNL DAAC 2011), as described further in the Data section of ESM. In general, however, the present modeling approach neither requires nor relies on this particular *ET* estimation method. If and where reliable data are directly available for *ET* over a whole long-term investigation period, then such time series both can and should be used in Eq. 2.

Soil parameter values used to evaluate *θ*_*uz*_ from equation S2 in ESM-Methods are listed in Table 2 with further details given in ESM-Data. Two scenarios of initial groundwater table *z*_gw−0_ = −1 m and *z*_gw−0_ = −2 m were used for realistic result exemplification (see ESM—Data for choice motivation), and a total soil depth of *z* = −2.5 m was used for the *θ*_*z*_ quantification (Eq. 3), similarly to conditions considered in Destouni and Verrot (2014).

**Table 2:** Soil parameter values used to evaluate equation S2 in Supplementary Material—Methods section for two contrasting soil types (selected values from Destouni 1991)

Parameter	Sand from Nontuna Arithmetic average over 1.8 m depth	Clay loam from Bro Arithmetic average over 1.8 m depth
*K*_s_ (m/s)	9.30 × 10^−5^	1.20 × 10^−5^
*θ*_ir_ (–)	0.02	0.15
*θ*_s_ (–)	0.45	0.40
*β* (–)	0.18	0.11

## Results and discussion

### Snow effects

**Fig. 2:**
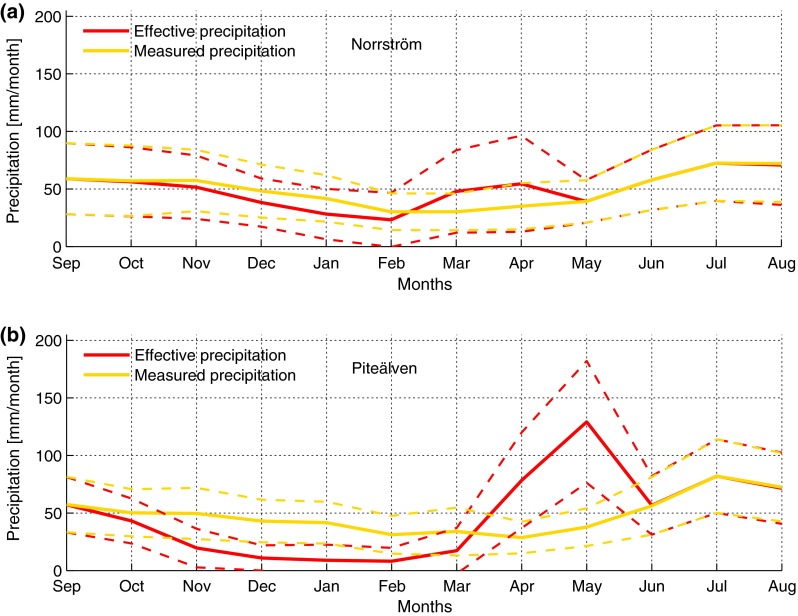
Average intra-annual distribution of monthly mean measured precipitation *P* (*yellow lines*) and mean effective precipitation *P*_eff_ (*red lines*) for the time period 1950–2009, in Norrström (**a**) and Piteälven (**b**). *Dashed lines* show one standard deviation from average values

The characteristics of *P*_eff_ and *P*, in terms of average intra-annual distribution over the whole study period (Fig. 2), show the effect of snow storage and melting in both study basins. The effect of snow storage is evident in smaller monthly *P*_eff_ than *P* values in winter, and the effect of snow melting is evident in greater monthly *P*_eff_ than *P* values in spring. As expected, the volume of water stored as snow in winter, and then released as liquid water in spring, is much greater in the cold Piteälven basin than in the warmer Norrström basin. Furthermore, the snow melting in Norrström is evenly spread over March–April, whereas in Piteälven, it is primarily spread over April–May with a pronounced melting peak in May. The snow storage season in winter is also longer in Piteälven (from October until March) than in Norrström (November until February).

The inter-annual variability in *P* and *P*_eff_, quantified by their respective standard deviation in Fig. 2, is further more or less similar over the months of an average year in Norrström, for both *P* and *P*_eff_. In Piteälven, the inter-annual variability of *P* is comparable to that in Norrström, but the inter-annual variability in *P*_eff_ is considerably smaller than that in *P* during winter, due to the regulating snow storage process.

### Temporal changes in each basin

**Fig. 3:**
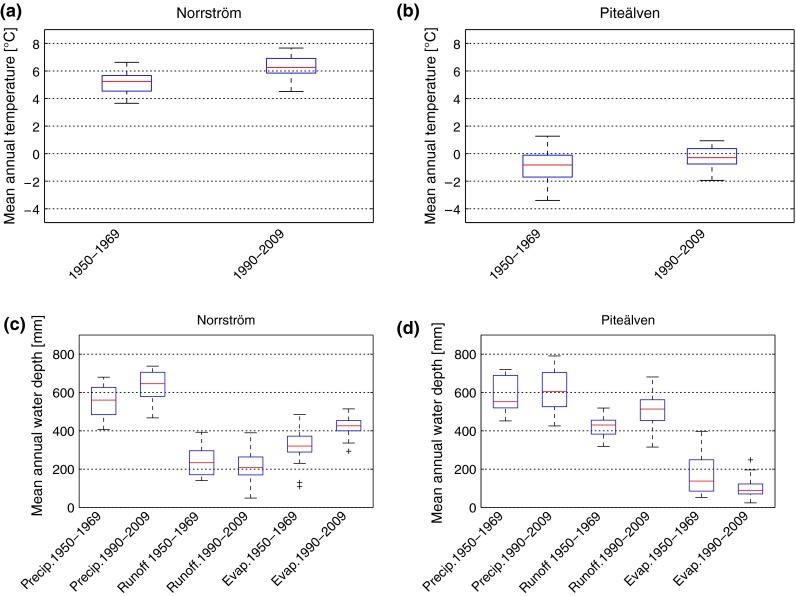
Boxplot of temperature (**a**, **b**) and other hydro-climatic variables: precipitation, runoff, evapotranspiration (**c**, **d**) for the periods 1950–1969 and 1990–2009. Results are shown for Norrström (**a**, **c**) and Piteälven (**b**, **d**)

Temporal change in different hydro-climatic variables is assessed by comparing the statistics of each variable across two 20-year climatic periods, from the beginning (1950–1969) to the end (1990–2009) of the whole study period (Fig. 3). Both basins have experienced warming (increased mean annual temperature *T*) of close to 1°C (somewhat less in Piteälven). Norrström has also experienced a relatively large increase in mean annual precipitation (*P*, by about 100 mm per year) while the runoff (*R*) has decreased over the same time (by 30 mm per year, due to even more increased *ET*; Destouni et al. (2013) have previously investigated increased *ET* in Norrström, explaining it as an effect of land- and water-use changes in this basin) (Fig. 3a). In Piteälven, *P* has only slightly increased between the two periods, while *R* has increased considerably more, implying that *ET* has here decreased between the periods (Fig. 3b). In both basins, inter-annual variability has most notably increased for *R* and decreased for *ET* (we refer to inter-annual variability as the 1.5 inter-quartile range on the boxplots).

**Fig. 4:**
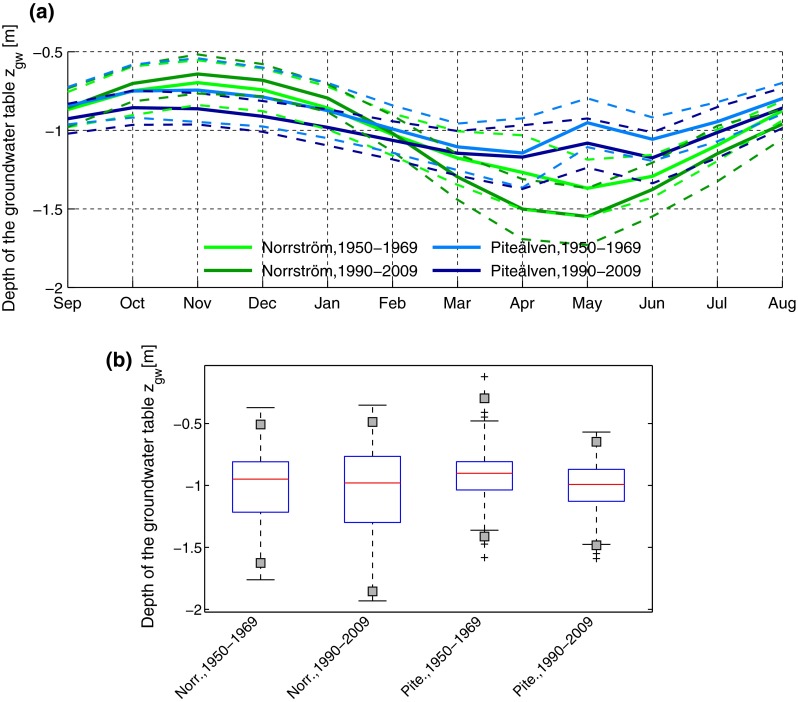
Average intra-annual distribution of groundwater table level *z*_gw_ (Eq. 2) in clay loam (**a**). Results are shown for two different time-periods and for the two study basins. *Dashed lines* show one standard deviation from average values. **b** Boxplot of monthly values. The *gray squares* represent the 1st and 99th percentiles

Results for the groundwater table position show that it has on average increased slightly in autumn and winter (September–February) in Norrström, while it has decreased notably during spring (March–May) (Fig. 4a). In Piteälven, however, the level of the groundwater table is significantly lower for the period 1990–2009, and for every month. Inter-annual variability has not changed in Piteälven while it has increased in Norrström (Fig. 4b). Piteälven has experienced less particularly high water table level events. In Norrström, the occurrence of more extreme events has not changed.

**Fig. 5:**
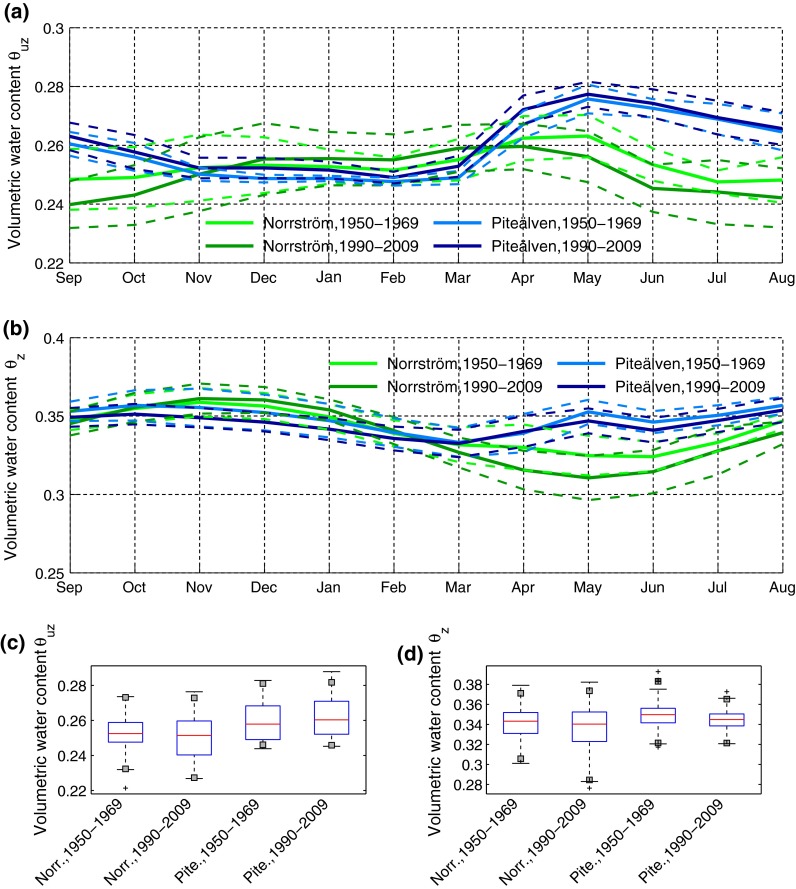
Average intra-annual distribution of monthly average water content *θ*_*uz*_ (equation S2 in Supplementary Material—Methods section) and monthly average water content *θ*_*z*_ (Eq. 3) over the depth *z* = −2.5 m (panels **a** and **b** respectively), for an initial value of the groundwater table *z*_gw−0_ = −1 m, in clay loam. Results are shown for two different time-periods and for the two study basins. *Dashed lines* show one standard deviation from average values. **c**, **d** Boxplots of monthly values of *θ*_*uz*_ (**c**) and *θ*_*z*_ (**d**). The *gray squares* represent the 1st and 99th percentiles

In terms of temporal changes to the water contents *θ*_*uz*_ and *θ*_*z*_ (Fig. 5), Piteälven has experienced a slight increase in mean unsaturated water content *θ*_*uz*_, reflecting the increase in runoff *R* in this basin (Fig. 3). In Norrström, where average annual *R* has decreased even though average annual *P* has increased (Fig. 3), there is on average less soil water available in the unsaturated zone from April to November, due to this basin’s increase in *ET* (Fig. 3c; see also Destouni et al. 2013), which is greatest during spring and summer, while in winter (December–March) *θ*_*uz*_ has slightly increased (Fig. 5a).

The changes in average water content *θ*_*z*_ over the greater soil depth −*z* = 2.5 m show decrease in the period March–August in Norrström and slight increase in winter (Fig. 5b) due to similar changes in groundwater level (Fig. 4a). In Piteälven, *θ*_*z*_ has decreased over the entire year (Fig. 5b) due to a similar change in groundwater level (Fig. 4a) and in contrast to the slight overall increase in *θ*_*uz*_ (Fig. 5a). The results are here exemplified for an original groundwater level −*z*_gw_ = 1 m, but are similar also for the scenario of −*z*_gw_ = 2 m (Destouni and Verrot 2014). Inter-annual variability has increased in Norrström for both *θ*_*uz*_ and *θ*_*z*_ (Fig. 5c, d), in consistency with the increased variability of both *R* (Fig. 2c) and groundwater level (Fig. 4b), as well as with previous results by Destouni and Verrot (2014), indicating increased occurrence of extreme conditions and particularly of dry events in this basin. In Piteälven, inter-annual variability has increased for *θ*_*uz*_ (due to increased variability of *R*; Fig. 3d) and has slightly decreased for *θ*_*z*_ (due to fewer extreme events of groundwater level; Fig. 4b).

### Comparison of basins along the north–south gradient

Spatial differences in *θ*_*uz*_ and *θ*_*z*_ (Fig. 5) between the warmer southern catchment (Norrström) and the colder northern one (Piteälven) can also be assessed for additional indications of climate effects on soil moisture. In the Piteälven drainage basin, the effect of the snow seasonality on *θ*_*uz*_ is considerable, with the added water input from the spring snow melt leading to a marked peak in *θ*_*uz*_ during April–May and a sustained high water content level in summer thereafter (Fig. 5a). In Norrström, without the added water input from spring snow melt, the soil dries considerably during spring and summer (May–August).

Overall, *θ*_*z*_ varies less over the year in Piteälven than in Norrström (Fig. 5b), reflecting a corresponding difference also in the groundwater table variation (Fig. 4). Similarly with *θ*_*uz*_, also *θ*_*z*_ decreases much more during summer in Norrström, without the spring snow melt, than in Piteälven, with its pronounced spring snow melt. Regarding inter-annual variability (Fig. 5c), this is smaller in Piteälven than in Norrström for *θ*_*uz*_ (due to smaller *R* variability in the former; Fig. 3c, d), as well as for *θ*_*z*_ (in the more recent period, due to the increase in groundwater level variability in Norrström, and to the smaller occurrence of extreme events in groundwater level in Piteälven, from the older to the recent; Fig. 4b).

### Comparative discussion

Previous studies have shown strong correlation between soil moisture variations and snow melting (Mahanama et al. 2012; Orth et al. 2013). Furthermore, the link used here between soil moisture and runoff (area-normalized stream discharge) has also been demonstrated in previous publications (Koster et al. 2010; Bales et al. 2011). Even though some studies report a dominant role of precipitation for soil moisture dynamics (Yin et al. 2014), this may not be so in cases, like the present ones, where runoff changes differ from precipitation changes, both due to snow-melt dynamics transforming the *P* dynamics into those of *P*_eff_ (Fig. 2) and due to evapotranspiration changes that can either counteract (Norrström, Fig. 3a) or enhance (Piteälven, Fig. 3b) *P* change effects on *R* and thereby also on soil moisture. Earlier studies have also shown low coupling between soil moisture and precipitation in northern regions where energy flux dynamics may be more important for soil moisture (Koster et al. 2004).

## Conclusion

The extension made here to analytical modeling of basin-scale long-term variability and change of soil moisture enables assessment of both spatial differences and temporal changes across a wide range of hydro-climatic conditions: from sub-zero to much warmer temperatures, in combination with various hydrological regimes. Intra-annual variability of soil moisture was found to differ considerably spatially between the two investigated basins, due to their temperature-related differences in snow storage-melting in an average year. With regard to temporal change, neither the long-term average nor the intra-annual variability of soil moisture has changed much in response to the hydro-climatic changes experienced so far in the two basins. Inter-annual soil moisture variability, however, has changed more notably in both basins, with the change in the variability of unsaturated water content, *θ*_*uz*_, and that of average water content over a fixed depth, *θ*_*z*_, being primarily determined by the change in inter-annual variability of water flux through the soil (*R* through *R*_eff_) and groundwater level, respectively.

Basin comparison along the north–south gradient shows that large spatial differences may not be realistically indicative of temporal climate change effects in a given region. Comparison of changes in average values and intra-annual variability with those in inter-annual variability of soil moisture shows that extreme-event statistics, reflected in the latter, may change considerably even under stable average and intra-annual variability conditions.

## Electronic supplementary material

Below is the link to the electronic supplementary material.

Supplementary material 1 (DOC 85 kb)

## References

[CR1] Aldahan A, Kekli A, Possnert G (2006). Distribution and sources of (129)l in rivers of the Baltic region. Journal of Environmental Radioactivity.

[CR2] Bales RC, Hopmans JW, O’Geen AT, Meadows M, Hartsough PC, Kirchner P, Beaudette D (2011). Soil moisture response to snowmelt and rainfall in a Sierra Nevada mixed-conifer forest. Vadose Zone Journal.

[CR3] Bosson E., U. Sabel, L.G. Gustafsson, M. Sassner, and G. Destouni. 2012. Influences of shifts in climate, landscape, and permafrost on terrestrial hydrology. *Journal of Geophysical Research*-*Atmospheres (1984*–*2012)* 117: D05.

[CR4] Braithwaite RJ, Zhang Y (2000). Sensitivity of mass balance of five Swiss glaciers to temperature changes assessed by tuning a degree-day model. Journal of Glaciology.

[CR5] Corradini, C. 2014. Soil moisture in the development of hydrological processes and its determination at different spatial scales. *Journal of Hydrology*. doi:10.1016/j.jhydrol.2014.02.051.

[CR6] Destouni G (1991). Applicability of the Steady state flow assumption for solute advection. Water Resources Research.

[CR7] Destouni G (1993). Stochastic modelling of solute flux in the unsaturated zone at the field scale. Journal of Hydrology.

[CR9] Destouni G, Verrot L (2014). Screening long-term variability and change of soil moisture in a changing climate. Journal of Hydrology.

[CR10] Destouni G, Jaramillo F, Prieto C (2013). Hydroclimatic shifts driven by human water use for food and energy production. Nature Climate Change.

[CR11] EEA 2012. European Environment Agency (EEA). Elevation map of Europe. Retrieved 21 February, 2014, from http://www.eea.europa.eu/.

[CR12] Graham W, Destouni G, Demmy G, Foussereau X (1998). Prediction of local concentration statistics in variably saturated soils: influence of observation scale and comparison with field data. Journal of Contaminant Hydrology.

[CR13] Granlund K, Rankinen K, Lepisto A (2004). Testing the INCA model in a small agricultural catchment in southern Finland. Hydrology and Earth System Sciences Discussions.

[CR14] Haylock, M.R., N. Hofstra, A.M.G. Klein Tank, E.J. Klok, P.D. Jones, and M. New. 2008. A European daily high resolution gridded data set of surface temperature and precipitation for 1950–2006. *Journal of Geophysical Research: Atmospheres**(1984*–*2012)* 113: D20119.

[CR15] Humborg C, Smedberg E, Blomqvist S, Mörth C-M, Brink J, Rahm L, Danielsson A, Sahlberg J (2004). Nutrient variations in boreal and subarctic Swedish rivers: Landscape control of land–sea fluxes. Limnology and Oceanography.

[CR16] Jaramillo F, Prieto C, Lyon SW, Destouni G (2013). Multimethod assessment of evapotranspiration shifts due to non-irrigated agricultural development in Sweden. Journal of Hydrology.

[CR17] Juston J, Seibert J, Johansson P-O (2009). Temporal sampling strategies and uncertainty in calibrating a conceptual hydrological model for a small boreal catchment. Hydrological Processes.

[CR18] Kininmonth, S., A. Bergsten, and Ö. Bodin. 2015. Closing the collaborative gap: Aligning social and ecological connectivity for better management of interconnected wetlands. *AMBIO* (Suppl. 1). doi:10.1007/s13280-014-0605-9. 10.1007/s13280-014-0605-9 PMC4289001 25576288

[CR19] Koster RD, Dirmeyer PA, Guo Z, Bonan G, Chan E, Cox P, Yamada T (2004). Regions of strong coupling between soil moisture and precipitation. Science.

[CR20] Koster RD, Mahanama SP, Livneh B, Lettenmaier DP, Reichle RH (2010). Skill in streamflow forecasts derived from large-scale estimates of soil moisture and snow. Nature Geoscience.

[CR22] Limbrick KJ, Whitehead PG, Butterfield D, Reynard N (2000). Assessing the potential impacts of various climate change scenarios on the hydrological regime of the River Kennet at Theale, Berkshire, south-central England, UK: An application and evaluation of the new semi-distributed model, INCA. Science of the Total Environment.

[CR23] Mahanama S, Livneh B, Koster R, Lettenmaier D, Reichle R (2012). Soil moisture, snow, and seasonal streamflow forecasts in the United States. Journal of Hydrometeorology.

[CR24] Molini, A., G.G. Katul, and A. Porporato. 2011. Maximum discharge from snowmelt in a changing climate. *Geophysical Research Letters* 38: L05402.

[CR25] Moor, H., K. Hylander, and J. Norberg. 2015. Predicting climate change effects on wetland ecosystem services using species distribution modeling and plant functional traits. *AMBIO* (Suppl. 1). doi:10.1007/s13280-014-0593-9. 10.1007/s13280-014-0593-9 PMC4288999 25576286

[CR26] Mörth C-M, Humborg C, Eriksson H, Danielsson A, Rodriguez Medina M, Lofgren S, Swaney DP, Rahm L (2007). Modeling riverine nutrient transport to the Baltic Sea: A large-scale approach. AMBIO.

[CR27] ORNL DAAC 2011. MODIS subsetted land products, collection 5. Available on-line Retrieved 5 May, 2013, from http://daac.ornl.gov/MODIS/modis.html. Oak Ridge, TN: Oak Ridge National Laboratory Distributed Active Archive Center (ORNL DAAC).

[CR28] Orth R, Koster RD, Seneviratne SI (2013). Inferring soil moisture memory from streamflow observations using a simple water balance model. Journal of Hydrometeorology.

[CR29] Quin, A., F. Jaramillo, and G. Destouni. 2015. Dissecting the ecosystem service of large-scale pollutant retention: The role of wetlands and other landscape features. *AMBIO* (Suppl. 1). doi:10.1007/s13280-014-0594-8. 10.1007/s13280-014-0594-8 PMC4288994 25576287

[CR30] Rankinen K, Kaste Ø, Butterfield D (2004). Adaptation of the integrated nitrogen model for catchments (INCA) to seasonally snow-covered catchments. Hydrology and Earth System Sciences.

[CR31] Rankinen K, Karvone T, Butterfield D (2004). A simple model for predicting soil temperature in snow-covered and seasonally frozen soil: model description and testing. Hydrology and Earth System Sciences.

[CR32] Rodriguez-Iturbe I, Entekhabi D, Bras RL (1991). Nonlinear dynamics of soil moisture at climate scales 1. Stochastic analysis. Water Resources Research.

[CR34] Russo D (1998). Stochastic analysis of flow and transport in unsaturated heterogeneous porous formation: Effects of variability in water. Water Resources Research.

[CR35] Schaefli B, Rinaldo A, Botter G (2013). Analytic probability distributions for snow-dominated streamflow. Water Resources Research.

[CR36] Seneviratne SI, Corti T, Davin EL, Hirschi M, Jaeger EB, Lehner I, Orlowsky B, Teuling AJ (2010). Investigating soil moisture–climate interactions in a changing climate: A review. Earth-Science Reviews.

[CR38] SMHI 2010. Swedish Meteorological and Hydrological Institute. Available on-line http://vattenwebb.smhi.se/.

[CR39] Tobin C, Nicotina L, Parlange MB, Berne A, Rinaldo A (2011). Improved interpolation of meteorological forcings for hydrologic applications in a Swiss Alpine region. Journal of Hydrology.

[CR40] Tobin C, Schaefli B, Nictina L, Simoni S, Barrenetxea G, Smith R, Parlange M, Rinaldo A (2013). Improving the degree-day method for sub-daily melt simulations with physically-based diurnal variations. Advances in Water Resources.

[CR41] Vehviläinen, B. 1992. Snow cover models in operational watershed forecasting. Helsinki: National Board of Waters and the Environment.

[CR43] Yin J, Porporato A, Albertson J (2014). Interplay of climate seasonality and soil moisture-rainfall feedback. Water Resources Research.

