# Supplementary material for: Screening variability and change of soil moisture under wide-ranging climate conditions: Snow dynamics effects
Source: Ambio. 2015 Jan 9;44(Suppl 1):6–16. doi: 10.1007/s13280-014-0583-y (PMC4289003; doi:10.1007/s13280-014-0583-y)
Supplement: Supplementary file 1 — Supplementary material 1 (DOC 85 kb) [file 13280_2014_583_MOESM1_ESM.pdf]

Supplementary Material

Screening variability and change of soil moisture under wide-ranging climate conditions: Snow dynamics effects

Lucile Verrot[1]*

Georgia Destouni[1]

[1] Department of Physical Geography & Quaternary Geology

Stockholm University

SE-106 91 Stockholm

Sweden

* Corresponding author :

E-mail: lucile.verrot@natgeo.su.se

Contents

- **Methods.** Details of θ_{uz} calculation method
- **Data**. Details of data used for the calculation of results

Methods

As in the Destouni and Verrot (2014) framework, we consider a soil depth of z_{ls} - z [L], with the vertical z-axis being positive upwards and z_{ls} being the land surface position along z. The generally variable groundwater table position at z_{gw} further determines the variable depth extent of the unsaturated zone, z_{ls} - z_{gw}, and that of the groundwater zone, z_{gw} - z, within the considered soil depth z_{ls} - z. The land surface position is then, for simplicity, set to z_{ls} = 0, and the dynamics of soil moisture are analyzed in terms of depth-average volumetric water content θ_{uz} [-] (referred to as water content in the following) in the unsaturated zone, and corresponding water content θ_z [-] over the whole considered soil depth -z.

Based on Brooks and Corey (1964), the unsaturated hydraulic conductivity K [LT^{-1}] in the unsaturated zone is further related to the unsaturated water content θ_{uz} as:

$$K(\theta_{uz}) = K_s \left(\frac{\theta_{uz} - \theta_{ir}}{\theta_s - \theta_{ir}} \right)^{1/\beta} \quad \text{(S1)}$$

where K_s [LT^{-1}] is the saturated hydraulic conductivity and θ_s [-] is the saturated soil water content, which can be assumed equal to porosity (Kumar 1999; Entekhabi et al. 2010). Moreover, θ_{ir} [-] is a residual soil water content, and β = 1/(3 + 2α) [-] and α [-] are characteristic soil texture parameters, linked to the pore size distribution of different soil types (Rawls et al. 1982; Saxton et al. 1986), with these Brooks-Corey (1964) parameters also being related to corresponding ones in the alternative constitutive relations of van Genuchten (1980) and vice versa (Morel-Seytoux et al. 1996). See further the Data section below for data used to evaluate the soil parameters in equation S1.

Utilizing the field-scale unsaturated flow and transport model of Dagan and Bresler (1979) and Bresler and Dagan (1981) for steady vertical gravity-driven flow, for which a unit hydraulic gradient may be assumed and the unsaturated hydraulic conductivity K in equation S1 can be approximately equated with average vertical soil water flux in the unsaturated zone, q [LT^{-1}],

further yields an approximate estimate of average water content θ_{uz} over the (variable) depth of the unsaturated zone as:

$$\theta_{uz} = \left(\frac{q}{K_s}\right)^{\beta} \left(\theta_s - \theta_{ir}\right) + \theta_{ir} \qquad \text{(S2)}$$

Destouni and Verrot (2014) explained in more detail how depth- and area-averaged unsaturated water content θ_{uz} can be estimated from equation S2 based on direct use of effective depth- area-averaged measures of time-dependent q and time-independent soil hydraulic properties K_s, θ_s, θ_{ir} and β. For the latter, both statistical (Destouni 1993) and deterministic (Destouni 1991) approaches to averaging their values over large scales and various depths of interest have been reported and used in previous studies.

Data

Calculation of θ_z, by use of z_{gw} from equation 2 in equation 3, requires at least monthly resolution of ET values along with corresponding P_{eff} and R_{eff} values. Such values do not exist from direct measurements over the whole investigation time period 1950-2009 for the two basins of study (as for most hydrological basins). In order to obtain monthly ET values for the whole long-term period 1950-2009, we followed the method developed by Destouni and Verrot (2014) of disaggregating annual values of ET, obtained from basin-scale annual water balance as ET_a=P_a-R_a, into corresponding monthly values $ET_{m,a}$ for each observation year a, as:

$$ET_{m,a} = ET_a \overline{\left(\frac{ET_{MODISm,a}}{ET_{MODISa}}\right)} \qquad \text{(S3)}$$

where ET_{MODISa} is annually aggregated evapotranspiration from directly observed monthly ET observations, $ET_{MODISm,a}$, for 2000-2010 from the MODIS-16 ET monthly product (ORNL DAAC, 2011), which is a global grid dataset with 0.05° x

0.05° resolution. Furthermore, $\overline{\left(\frac{ET_{MODISm,a}}{ET_{MODISa}}\right)}$ is the mean value of the monthly to annual evapotranspiration ratio for month m over the MODIS dataset period 2000-2010.

In this context, we note that the assumption of ET_a=P_a-R_a allows for basin-scale water storage changes within a year but not between years. In general, however, the present modeling approach neither requires nor relies on this constraining assumption. If and where independent long-term data is available for ET, then such time series both can and should be used in equations 1 and 2, thus allowing for basin-scale storage changes also inter-annually. However, for studies where ET_a=P_a-R_a has to be used, we also note that its applicability has been checked by Jaramillo et al. (2013) through direct comparison with other ET estimation methods, showing that it may be sufficiently accurate and thus useful for the type of long-term variability and change screening that is the main scope of the present study. Furthermore, even with the assumption ET_a=P_a-R_a, the overall groundwater level calculated by equation 2 can to some degree change from year to year, due to the difference of P_{eff} from measured P_a. Moreover, even if the groundwater table, as a consequence of balanced water fluxes, starts and ends each hydrological year at the same position, it can still have average level between these annual time points that differs among years.

Soil parameter values are further needed to evaluate θ_{uz} in equation S2. As concrete examples of relevant area-depth-averaged values we here used measurement-based results reported in Destouni (1991) for depths sampled down to 1.8 m depth below the surface. We considered then in our calculations values for two contrasting soil types (sand and clay loam) from different locations (Nontuna and Bro, respectively) within the Norrström drainage basin (Table 2). As the focus of the present study is on hydro-climatic and snow dynamics effects on soil moisture, rather than on the effect of soil characteristics, we used for result exemplification the same soil parameter values for both the Norrström and the Piteälven basin. In result figures, only results for clay loam are shown; in fact, calculated soil moisture dynamics (variability and change patterns) were similar between soil types even though absolute soil moisture

levels differed. These dynamics similarities and overall level differences are consistent with findings reported by Destouni and Verrot (2014).

A specific initial groundwater table position $z_{gw\text{-}0}$ is also needed but not known for the present long-term study period in Norrström and Piteälven. Mesic-Moist areas (soils in which the groundwater table is located between 0.5 m and 1 m below the surface) and Mesic areas (soils in which the groundwater table is located between 1 m and 2 m below the surface) are the most frequent ones in both the Norrström and the Piteälven basin (SLU 2002; von Arnold et al. 2005). For result exemplification, we therefore used both $z_{gw\text{-}0}$ = -1 m and $z_{gw\text{-}0}$ = -2 m when evaluating groundwater table position z_{gw} (equation 2).

References

Bresler, E., and G. Dagan. 1981. Convective and pore scale dispersive solute transport in unsaturated heterogeneous fields. *Water Resources Research* 17: 1683–1693.

Brooks, R.H. and A.T. Corey. 1964. Hydraulic properties of porous media. *Hydrology Papers*, Colorado State University.

Dagan, G., and E. Bresler. 1979. Solute dispersion in unsaturated heterogeneous soil at field scale: I. Theory. *Soil Science Society of America Journal* 43: 461–467.

Destouni, G. 1991. Applicability of the Steady State Flow Assumption for Solute Advection. *Water Resources Research*. 27: 2129–2140.

Destouni, G. 1993. Stochastic modelling of solute flux in the unsaturated zone at the field scale. *Journal of Hydrology* 143: 45–61.

Destouni, G., and L. Verrot. 2014. Screening long-term variability and change of soil moisture in a changing climate. *Journal of Hydrology* 516: 131–139. doi:10.1016/j.jhydrol.2014.01.059.

Entekhabi, D., R.H. Reichle, R.D. Koster, and W.T. Crow. 2010. Performance metrics for soil moisture retrievals and application requirements. *Journal of Hydrometeorology* 11: 832–840.

Jaramillo, F., C. Prieto, S.W. Lyon, and G. Destouni. 2013. Multimethod assessment of evapotranspiration shifts due to non-irrigated agricultural development in Sweden. *Journal of Hydrology* 484: 55–62.

Kumar, P. 1999. A multiple scale state-space model for characterizing subgrid scale variability of near-surface soil moisture. *IEEE Transactions on Geoscience and Remote Sensing* 37: 182–197.

Morel-Seytoux, H. J., P.D. Meyer, M. Nachabe, J. Tourna, M.V. Genuchten, and R.J. Lenhard. 1996. Parameter equivalence for the Brooks-Corey and van Genuchten soil characteristics: Preserving the effective capillary drive. *Water Resources Research* 32: 1251–1258.

ORNL DAAC 2011. MODIS subsetted land products, collection 5. Available on-line (http://daac.ornl.gov/MODIS/modis.html) from Oak Ridge National Laboratory Distributed Active Archive Center (ORNL DAAC), Oak Ridge, Tennessee, U.S.A (Retrieved May 5, 2013).

Rawls, W.J., D.L. Brakensiek, and K.E. Saxton. 1982. Estimation of soil water properties. *Transactions of the American Society of Agricultural Engineers* 25: 1316–1320.

Saxton, K.E., W. Rawls, J.S. Romberger, and R.I. Papendick. 1986. Estimating generalized soil-water characteristics from texture. *Soil Science Society of America Journal* 50: 1031–1036.

SLU 2002. Department of Forest Soils, Swedish University of Agricultural Sciences, Uppsala (SLU). Current results from the Swedish survey of forest soils and vegetation (SK), retrieved October 2, 2013, from http://www-markinfo.slu.se.

van Genuchten, M.T. 1980. A closed-form equation for predicting the hydraulic conductivity of unsaturated soils. Soil Science Society of America Journal 44: 892–898.

von Arnold, K., B. Hånell, J. Stendahl, and L. Klemedtsson. 2005. Greenhouse gas fluxes from drained organic forestland in Sweden. Scandinavian Journal of Forest Research 20: 400–411.
